# The EU chemicals strategy for sustainability: an opportunity to develop new approaches for hazard and risk assessment

**DOI:** 10.1007/s00204-022-03313-2

**Published:** 2022-05-11

**Authors:** Stefan Scholz, Werner Brack, Beate I. Escher, Jörg Hackermüller, Matthias Liess, Martin von Bergen, Lukas Y. Wick, Ana C. Zenclussen, Rolf Altenburger

**Affiliations:** 1 Department of Bioanalytical Ecotoxicology, Helmholtz Centre for Environmental Research-UFZ, Permoserstr. 15, 04318 Leipzig, Germany; 2 Faculty Biological Sciences, Goethe University Frankfurt, Max-von-der-Laue-Straße 13, 60438 Frankfurt, Germany; 3 Department of Cell Toxicology, Helmholtz Centre for Environmental Research-UFZ, Permoserstr. 15, 04318 Leipzig, Germany; 4 Center for Applied Geoscience, Eberhard Karls University of Tübingen, 72076 Tübingen, Germany; 5 Department Computational Biology, Helmholtz Centre for Environmental Research-UFZ, Permoserstr. 15, 04318 Leipzig, Germany; 6 Faculty of Mathematics and Informatics, University of Leipzig, 04109 Leipzig, Germany; 7 Department Systems Ecotoxicology, Helmholtz Centre for Environmental Research-UFZ, Permoserstr. 15, 04318 Leipzig, Germany; 8 RWTH Aachen University, Institute for Environmental Research (Biology V), Aachen, Germany; 9 Department Molecular Systems Biology, Helmholtz Centre for Environmental Research-UFZ, Permoserstr. 15, 04318 Leipzig, Germany; 10 Faculty of Life Sciences, Institute of Biochemistry, University of Leipzig, Brüderstraße 34, 04103 Leipzig, Germany; 11 Department Environmental Microbiology, Helmholtz Centre for Environmental Research-UFZ, Permoserstr. 15, 04318 Leipzig, Germany; 12 Department Environmental Immunology, Helmholtz Centre for Environmental Research-UFZ, Permoserstr. 15, 04318 Leipzig, Germany; 13 Perinatal Immunology, Saxonian Incubator for Clinical Translation (SIKT), Medical Faculty, Leipzig University, Philipp-Rosenthal-Str. 55, 04103 Leipzig, Germany; 14 Department of effect directed analysis, Helmholtz Centre for Environmental Research - UFZ, 04318 Leipzig, Germany

**Keywords:** European Union, Sustainable chemistry, Hazard assessment, Risk assessment

The EU chemicals strategy for sustainability (CSS) (EC 2020b) sets policy goals regarding future ambitions for the safe use and management of chemicals and aims at “chemicals [to be] used more safely and sustainably, promoting that chemicals having a chronic effect for human health and the environment—substances of concern—are minimised and substituted as far as possible, and phasing out the most harmful ones for non-essential societal use, in particular in consumer products”. To achieve this goal, the EU chemicals strategy lists several areas requiring future actions with potential consequences on procedures of chemical regulations currently in place.

The CSS is motivated by public concerns and scientific findings regarding the potential adverse impacts of chemicals on the environment and human health. It has been intensively discussed within the scientific community. In many reviews, the CSS received positive feedback regarding its goals and ambitions. For instance, the CSS was considered as “the first regional framework aiming to address chemical pollution in a holistic manner” (van Dijk et al. 2021) or it was appreciated that “prevention and reduction of pollution [was] on the same political level as the protection of climate and biodiversity” (Conrad et al. 2021). However, certain aspects of the CSS were more critically commented, such as the use of terminology (zero pollution, non-toxic, toxic-free) or the lack of a strategic plan how to implement the zero pollution goal (Herzler et al. 2021; van Dijk et al. 2021).

Here, we particularly respond to the opinion expressed in a guest editorial by members of the Federal Institute of Risk Asssement (BfR) of Germany (Herzler et al. 2021) and a supportive letter to the editor (Barile et al. 2021). Herzler et al. (2021), in addition to the critical assessment of terminology, expressed specific concerns that the CSS would negatively affect current risk assessment procedures. They argued that current schemes of risk assessment are sufficiently science based and performing generally well with regard to the goal of protection of human populations from chemical impacts. The editorial further denies that there is sufficient scientific evidence to justify additional regulatory measures as suggested by the CSS, and criticises its focus on hazard-based assessment of chemicals and their potential mixture effects.

We provide a different, but also science-based perspective to the CSS. We consider the CSS primarily as a policy statement that asks for science-based approaches to meet the challenges of its implementation. Progress in protecting human health and the environment from undue chemical impacts requires a dialogue of regulatory agencies with scientists, and other stakeholders, taking into account past experiences as well as scientific advances. The CSS thus provides an opportunity to revise, modernise, and improve current hazard and risk assessment procedures based on sound science and pursuing ambitious goals. In this respect, we would like to complement the view of Herzler et al (2021). As it is beyond the scope of this letter to provide an in-depth review of the scientific literature, we rather aim at fostering a constructive and forward-looking scientific debate for best implementation of the CSS goals. We thereby will focus on the following aspects:

1. Terminology of a non-toxic environment and the role of hazard versus risk assessment.
2. Evidence for sufficient protection of human health regarding chemical exposure by existing regulation.
3. Evidence for mixture effects and the need to incorporate mixture toxicity in chemical regulation.
4. Fostering the scientific debate to consider and address worries of the population.

## Terminology of a non-toxic environment and the role of hazard versus risk assessment

The CSS terminologies “zero pollution”, “non-toxic” or “toxic-free” are not self-explanatory and are not defined unambiguously. This may lead to misunderstandings that hamper the implementation of the CSS goals. This issue was previously identified by a CSS opinion paper indicating that such terminology was rather “reflecting the opinion of the society, as many Europeans are concerned about the environmental impact of chemicals present in everyday products” (van Dijk et al. 2021). The CSS, however, also states that chemicals should be” produced and used in a way that maximises their contribution to society including achieving the green and digital transition, while avoiding harm”. This implies that zero pollution may not be the same as zero exposure. In this line of thinking, van Dijk et al. (2021) proposed that a toxic-free environment should be interpreted as “an environment in which all chemicals can be emitted as a result of human activities, but in low concentrations so that no adverse effects to organism occur”. The definition of van Dijk et al. (2021) is also in line with common text-book terminology of exposure science and toxicology considering the dose of a chemical as relevant for its classification as a pollutant or toxicant.

Chemical risk assessment is typically based on the assumption that human and environmental exposure to hazardous chemicals can be predicted and exposures to dangerous levels can be avoided. In contrast to risk assessment, a hazard-centred assessment (i.e. an assessment based on the intrinsic capacity of a chemical to elicit adverse effects) can be useful in cases where little exposure information is available and prospective assessment is required. This is already current practice in certain regulations; prominent examples are the PBT (persistent, bioaccumulative, toxic) assessment and the classification of compounds as SVHC [substances of very high concern—carcinogenic, mutagenic or reproductive toxicity (CMR) or endocrine disrupting chemicals (EDC)] in REACH. The precautionary principle of EU chemical regulation is the major rationale to base certain regulatory decisions on hazard assessment alone if long-term effects cannot reliably be predicted. For persistent chemicals, a widespread environmental pollution can lead to accumulation in biota. Once in the environment, persistent chemicals cannot be removed, and, hence, this compound property justifies a hazard-based assessment. For SVHCs such as EDCs, the risk of health effects at even low doses and the uncertainty of predicted exposures may be considered too high. Consequently, the classic approach of comparing exposure levels with predicted no-effect concentrations in environmental and derived no-effect levels in human health risk assessment may not be appropriate. There are options to improve hazard-based assessments: for instance, a hazard-based assessment may be considered in a first-tier approximation which provides a scientific incentive to identify and select appropriate tools, including alternative, non-animal testing approaches, for better characterisation of such compounds. This has been discussed, e.g. for endocrine disruption (Natsch 2021).

A hazard-based approach could also be sufficient when discussing the replacement of compounds for which health risks have been identified—to avoid regrettable substitutions by compounds with similar properties (Zimmerman and Anastas 2015). This is of particular relevance for persistent chemicals that may accumulate eventually to hazardous concentrations. Finally, a hazard-based assessment is also relevant in the context of sustainable circular use of chemicals to avoid that hazardous chemicals are unintentionally recycled into new articles resulting in unexpected human exposure (Wang and Hellweg 2021) as, e.g. demonstrated for bisphenols used in thermal paper, which is recycled to a wider range of paper products (Liao and Kannan 2011). The ambition for a circular economy as established in the Circular Economy Action Plan by the European Commission (EC 2020c) may further help to avoid the exposure to additional hazardous chemicals in products.

## Evidence for sufficient protection of human health regarding chemical exposure by existing regulation

In traditional toxicology apical end points, such as survival, growth or number of offspring represent the main end points are to estimate the potency of a chemical to cause adverse effect and to calculate a risk with respect to an expected exposure condition. With regard to these apical end points, it may seem tempting to consider the increase in life expectancy or the growth of the world populations as possible indicators of successful chemical risk assessments as Herzler and colleagues (2021) argue. Indeed, from the perspective of the increase of our standard of living, health care, improved sanitation and nutrition, an impact of chemicals on human health seems less obvious. But studies with twins suggested that environmental—including exposure to chemicals—rather than genetic determinants—constitute the major cause for a range of chronic diseases (Rappaport 2016). The Lancet Commission on pollution highlighted the principal contribution of pollution on health, as expressed by an estimated 9 million premature deaths worldwide in 2015 (Landrigan et al. 2018). To a large extent, in about 7.8 million, these pollution-associated premature deaths were attributed to air pollution (particles, ozone) or unsafe water sanitation. Exposure to chemicals not related to air pollution was associated to more than a million premature deaths. However, to which extent chemical exposure via consumer products, food or drinking water affect health is less clear. The WHO and others proposed that various sources of chemical exposure need to be associated with negative health outcomes (Naidu et al. 2021; Prüss-Ustün et al. 2011; WHO 2016). Potential human health effects due to exposure to chemicals have, for instance, been discussed for neurodevelopmental disorders (Bennett et al. 2016), obesity (Mohanto et al. 2021) or male reproductive health (Foresta et al. 2018; Pollard et al. 2019; Wu et al. 2022).

In modern toxicology and health sciences, it is well established that humans are exposed to a large number of chemicals whereby their effects on health are often uncertain. Chemicals may impact on the health status of organisms at concentrations that are below those causing consented adverse apical effects. However, addressing and quantifying the association of exposures with the prevalence of common and non-communicable diseases is still a major challenge for research. This is illustrated by the example of obesity. For obesity, about two decades of gene wide association studies could only associate 40–70% of the risk for developing elevated BMI to a genetic background (Locke et al. 2015), leaving 30–60% unexplained. Environmental factors like plasticisers, which do affect the differentiation of adipocytes, are suspected to play a role. In several metastudies (Goodman et al. 2014; Ribeiro et al. 2019), however, no clear results were found, leaving the question to which degree plasticisers contribute to the aetiology of obesity on the population level open and thus supporting the demand for further investigations.

Acknowledging the challenge in associating chemical and disease, in our view, is not equal to denying any role, but rather may call for precautionary approaches until the safety of chemicals can be ascertained. Novel science-based approaches are needed here, to quantify whether and to which extent chemicals contribute to disease outcomes. For instance, integration of exposure assessment, epidemiological evidence and experimental approaches can be used to establish links between exposure and negative health outcomes. This was shown in a recent study (Caporale et al. 2022) that established mechanistic and correlative evidence for an association of in utero exposure to mixtures of endocrine disruptors and learning disabilities in children.

An extended focus on health implications, diseases or non-apical effect proxies also call for development, testing and application of new approach methods (NAMs) in the widened screening and assessment of chemicals. NAMs comprise computational, omics approaches and alternative test systems, of which the end points are conceptually linked to molecular initiating and key events of adverse outcome pathways. There is an increasing demand for application of NAMs, not only motivated by the intention to reduce animal testing, but also by concerns of weak predictivity of established animal test models. Moreover, there is a need to increase the capacity of chemical testing (Fentem et al. 2021; Parish et al. 2020) to keep up with chemical innovation. It has been argued that the increasing rate and diversity of production of chemicals exceed societies’ ability to efficiently conduct safety-related assessments and monitoring and thus transgress the safe operating space of the planetary boundaries for novel entities (Persson et al. 2022). One can argue that application of NAMs with increasing automation has the potential to overcome these limitations, so that testing capacity will not remain a bottleneck. The scientific challenge for the application of NAMs is their interpretation within regulatory frameworks. The following questions have to be answered for regulatory implementation: (i) What can be regarded as sufficient evidence from NAM-based observations to infer a risk of adverse health effects? (ii) How do NAMs perform in comparison to other pragmatic approaches proposed for regulation such as the threshold of toxicological concern (TTC)? and (iii) How can we identify and select suitable NAMs and define regulatory thresholds for restriction of use or banning of chemicals? The upcoming European Initiative “European Partnership for Chemicals Risk Assessment under Horizon Europe (PARC)” with its composure of science-oriented institutions from the regulatory and public research sphere offers the unique opportunity to serve as a framework to develop and evaluate novel concepts for NAMs-based assessments useful for regulatory decision making (EC 2020a).

## Evidence for mixture effects and the need to incorporate mixture toxicity effects in regulation

A recent analysis of public inventories estimated approximately 355 000 chemical that have been registered for production and use, with approximately 69 000 chemicals in commerce (Wang et al. 2020). The production of chemicals has doubled from 2000 to 2015 (Persson et al. 2022) and is expected to double again from 2015 to 2030 (EC 2017; UNEP 2019).

These sheer numbers support that exposure of environmental organisms and humans to chemical mixtures requires accelerated consideration in the assessment of chemicals (Escher et al. 2020). There is experimental long-standing proof-of-principle evidence that mixture exposure can provoke combined effects, even if concentrations of individual compounds occur below their individual effect thresholds (Altenburger and Greco 2009; Kortenkamp et al. 2009). Mathematical and toxicodynamically founded models were developed, supporting and explaining the experimental findings (Kortenkamp et al. 2007; Rider et al. 2018).

Furthermore, lessons learnt in ecotoxicology could be instructive. Monitoring the species abundance in our freshwaters has indicated that the impact of chemical exposure in the environment is larger than expected and that apparently the current approach for prospective risk assessment may not have been sufficient (Liess et al. 2021; Malaj et al. 2014). Also, despite prospective chemical risk assessment and measures under the European Water Framework Directive to improve the status of environmental quality, many surface waters were assigned a moderate to bad ecological status. Among other factors, this failed good status was largely attributed to chemical pollution (EEA 2018; Lemm et al. 2021).

One may dispute the way how chemical mixtures are considered in chemical regulation and whether the use of a mixture assessment factor is a universal or the optimal solution for a specific case. Yet, the application of safety factors is an accepted regulatory practice in accounting for uncertainty in other areas such as cross-species, or exposure duration extrapolation. The CSS provides a mandate to develop and improve assessment of chemicals with regard to mixture exposure. Central to improve mixture assessment is an improved exposure assessment to identify relevant mixture exposure prospectively. For retrospective assessment and monitoring, comprehensive assessment of human exposure to mixtures using advanced technologies of chemical analysis has been just begun, and more systematic assessment of the human exposome supported by advanced detection technologies will likely demonstrate even more complex exposure situations than currently considered in risk assessment (Huhn et al. 2021). Alternatively in the future, one may use a whole-mixture approach, where mixtures are extracted from (human) samples and their effects quantified with high-throughput cell-based bioassays (Vinggaard et al. 2021). We agree that the estimation of the contribution of chemicals to diseases in humans is not an easy task given the importance of intrinsic factors such as genetic predisposition and transgenerational effects or socioeconomic factors, nutrition, air quality, access to green space, physical activity and many more. Hence, research to understand the effect of mixtures and appropriate consideration of mixtures within chemical regulations is needed and has been called for by various EU scientific advisory boards (SCHER 2012) almost a decade ago.

## Fostering the scientific debate to consider and address worries of the population

The EU chemical sustainability strategy states that “84% of Europeans are worried about the impact of chemicals present in everyday products on their health, and 90% are worried about their impact on the environment”. The editorial of Herzler et al. (2021) criticises that the concerns may partially be subjective, lacking scientific evidence, and hence should not drive decisions on chemical regulations. In our perspective, the CSS represents primarily a policy document, which by its nature also considers the actual risk perception of European citizens for health impacts of chemicals. However, the mandate defined by the CSS sets the state for intensified discussion between the regulatory agencies and related scientific research institutions in Europe for scientifically defined improvements of hazard and risk assessment. As outlined above, there is also scientific evidence for (potential) impacts of chemical pollution on human health, albeit the exact magnitude of the contribution may often not be known. Therefore, the CSS should be considered as a political mandate and a motivation to close knowledge gaps, effectively communicate new findings to the public and improve regulations minimising the risk of chemical exposure for human and environmental health.

## Conclusions

We have evidence of a multitude of chemicals being present in the environment and in our bodies and that mixture exposure indeed matters. This knowledge needs to be deepened, and the quantitative contribution of chemicals to compromised health should be better described and translated into regulatory action. As indicated in a scientific opinion paper of the German Federal Environmental Agency (Conrad et al. 2021), the CSS goals may be considered as a moving target. For increasing scientific evidence and improved method for detection and assessment of chemicals, development of new technologies require innovative regulatory, technological and societal reactions. We should be flexible and prepared to take up the scientific challenges and collaborate productively with regulatory institutions to address the identified challenges and modernise chemical risk assessment. This is also in line with the concern of many scientists that chemical pollution and the wide range of adverse effects on human and ecosystem health demand additional efforts on a global scale (Brack et al. 2022; Wang et al. 2021). We see the CSS as a European strategy that, in concert with other initiatives, may open new opportunities to minimise hazardous chemical pollution and thus risks to human health and ecosystems.
